# Long-term cost-utility analysis of remote monitoring of older patients with pacemakers: the PONIENTE study

**DOI:** 10.1186/s12877-020-01883-3

**Published:** 2020-11-16

**Authors:** Rafael Jesus Bautista-Mesa, Antonio Lopez-Villegas, Salvador Peiro, Daniel Catalan-Matamoros, Emilio Robles-Musso, Remedios Lopez-Liria, Cesar Leal-Costa

**Affiliations:** 1 Management Unit, Hospital de Poniente, El Ejido, Almeria, Spain; 2 Social Involvement of Critical and Emergency Medicine, CTS–609 Research Group, Hospital de Poniente, El Ejido, s/n, 04700 Almeria, Spain; 3 Health Services Research Unit, FISABIO-PUBLIC HEALTH, Valencia, Spain; 4 Department of Communication Studies, University Carlos III of Madrid, Madrid, Spain; 5 Health Sciences CTS–451 Research Group, University of Almería, Almería, Spain; 6 Pacemaker Unit, Intensive Care Unit, Hospital de Poniente, El Ejido, Almería, Spain; 7 Department of Nursing Science, Physiotherapy and Medicine, Hum–498 Research Team, Health Research Centre, University of Almería, Almería, Spain; 8 Nursing Department, University of Murcia, Murcia, Spain

**Keywords:** Cost-utility, Pacemakers follow-up, Quality-adjusted life years, Remote monitoring, Telemedicine

## Abstract

**Background:**

Cost-effectiveness studies on pacemakers have increased in the last years. However the number of long-term cost-utility studies is limited. The objective of this study was to perform a cost-utility analysis comparing remote monitoring (RM) versus conventional monitoring (CM) in hospital of older patients with pacemakers, 5 years after implant.

**Methods:**

Under a controlled, not randomized, nor masked clinical trial, 83 patients with pacemakers were initially selected. After five years of follow-up, a total of 55 patients (CM = 34; RM = 21) completed the study. A cost-utility analysis of RM in terms of costs per gained quality-adjusted life years (QALYs) was conducted. The costs from the Public Health System (PHS) as well as patients and their relatives were taken into account for the study. The robustness of the results was verified by the probabilistic analyses through Monte-Carlo simulations.

**Results:**

After a five-year follow-up period, total costs were lower in the RM group by 23.02% than in the CM group (€274.52 versus €356.62; *p* = 0.033) because of a cost saving from patients’ perspective (€59.05 versus €102.98; *p* = 0.002). However, the reduction of in-hospital visits derived from RM exhibited insignificant impact on the costs from the PHS perspective, with a cost saving of 15.04% (€215.48 vs. €253.64; *p* = 0.144). Costs/QALYs obtained by the RM group were higher as compared to the CM group, although there were no significant differences. The incremental cost-effectiveness ratio of CM in comparison to RM became positive (€301.16).

**Conclusions:**

This study confirms RM of older patients with pacemakers appears still as a cost-utility alternative to CM in hospital after 5 years of follow-up.

**Trial registration:**

ClinicalTrials.gov: (Identifier: NCT02234245). Registered 09 September 2014 - Prospectively registered.

## Background

As per the norms of the professional practice guidelines, revisions at a frequency of every 3–12 months are essential for older patients implanted with pacemakers (PM) to evaluate the patient’s clinical status as well as the PM functioning and, in case, its reprogrammation [1, 2]. Remote monitoring (RM), an alternative to conventional monitoring (CM) appeared two decades ago [1, 3, 4] in hospitals. It was implemented to balance time and costs requirements not only from the Public Health System (PHS) [5] perspective but also from patients and their relatives’ perspective [4, 6].

Existing research has demonstrated RM as effective as hospital monitoring [1, 6–9], in terms of safety [3] and reduction of the time to clinical decision and intervention. It provides rapid detection of cardiovascular events [10] or device malfunction as well as reduces inappropriate shocks and spares implantable cardioverter defibrillators’ batteries [9, 11]. RM is also successful in improving patient’s satisfaction and gaining their confidence. RM empowers patients for self-management of their conditions [12, 13]. Visits to the hospitals become limited only for relevant device alerts [10, 14]. The number of unscheduled in-hospital visits, the assistance of emergency services, and hospitalizations are reduced by RM [5, 15]. On the contrary, it is essential to educate and train the patients properly regarding the purpose and benefits of RM, its usage, and limitations [16] so that their preferences for in-hospital follow-up could be reduced [17]. This is related to social acceptance of telehealth, one of the three dimensions jointly utility and usability that would help predict how a new technology will be utilized with patients [18, 19].

Cost-effectiveness analyses are employed to compare the effectiveness of new interventions against their incremental cost to be considered in the medical decision-making process as well as in public policy [14, 20]. The last few years have witnessed an upsurge in the number of cost-effectiveness studies on cardiovascular implantable electronic devices [8, 9, 21–25]. However, there have been only a few studies that analyzed cost-utility in terms of quantity and health-related quality of life (HRQL) of older patients, especially in the long-term [5, 10, 14, 26–29].

Early results from the cost-utility analysis performed in the PONIENTE study after a 12-months follow-up period [30], confirmed that RM of patients with PMs is a cost-saving and cost-utility alternative in comparison to conventional follow-up in a hospital. This article is an extension of the PONIENTE study to a five-year follow-up period, with the aim of evaluating cost-utility (in terms of cost per quality-adjusted life-years gained, (QALYs) of long-term RM of older patients with pacemakers.

## Methods

The PONIENTE study is a controlled, neither randomized nor masked, single-center clinical trial, designed to compare RM of older patients with pacemakers (active group) to the CM in hospital (control group), with 5 years of follow-up from the initial date of pacemakers implantation (between October 2012 to November 2013).

### Intervention description

The PONIENTE trial was conducted on older patients (81 years old on average) recruited in the Poniente Hospital (Almeria–Spain) implanted with commercially available pacemakers equipped with the Medtronic CareLink® Network. As previously reported [7, 30], 83 patients were initially included in the cost-utility study. Other relevant information about the PM technology, as well as its functioning mode, was mentioned in a previous paper [31]. Effectiveness results (functional capacity, event detection, and workload) were also depicted in an earlier paper [7]. Based on the study protocol, patients were followed-up for a period of 5 years after the implant date through scheduled in-hospital visits or remote monitoring. One month after the implant date, the physician scheduled an in-office visit with the patients, explained the characteristics of both types of monitoring and tried to determine which type of monitoring (remote or conventional) was better suited to the patient characteristics. Patients included in both groups were managed according to the usual practices of the hospital, abiding by the international guidelines with follow-up visits scheduled under physician’s criteria and regulation from the Spanish Health Authority. The number of visits to the hospital (CM = 7.49 and RM = 4.38 on average in the total 5-years of follow up period) and/or transmissions from home (RM = 6.62 on average in the total 5-years of follow up period) for each patient and year depended on the type of pacemaker implanted and patient characteristics.

Patients who opted for the RM were provided with a device (Medtronic Carelink Home Monitor) for data transmissions through a standard phone line from home to a central database, where data were processed and made accessible to physicians and healthcare team. It does not provide any additional therapeutic capabilities to the devices. The corresponding PM parameters were programmed, the use of the device as well as the protocol for sending data was explained and, finally, the service from the supplier company was requested by the physicians. Following the norms of the international consensus on the monitoring of cardiovascular implantable electronic devices [1], PM specifications, and physicians’ criteria, patients under RM were asked to submit data at different time points. No further in-hospital visits were scheduled for patients in the RM group. On the contrary, they were connected over the telephone and referred for an in-office visit, if data transmissions detected any device dysfunction or cardiovascular event. Data collected from all remote transmissions were scrutinized daily by the administrative staff and reviewed by the physicians through password-protected remote access.

### Costs analysis

Costs analysis was studied from two different perspectives (Table 1): firstly, costs incurred by the hospital, funded by the Spanish PHS; secondly, social costs afforded by the patients and their caregivers.

**Table 1:** Variables definition and costs included in the study

Perspective	Variables	Unitary cost (UC)	Monitoring^**c**^	Cost per patient model^**d**^
Public Health System^a^	Physician labor costs	€0.54/min	CM	NVH x PTH x UC
			RM	NRT x PTR x UC
	Administrative labor costs	€0.27/min	CM	NVH x ATH x UC
			RM	NTH x ATR x UC
	Consultation-room	€0.029/min	CM	NVH x PTH x UC
			RM	
	Ambulance	€1.13/km	CM	NVHA x UC
			RM	
Patients and relatives^b^	Transportation (round trip)	Taxi (ranged from €14.50 to €142.10); Bus (ranged from €2.60 to €7.43); Private car (€0.19/km)	CM	(NVH – NVHA) x UCIT
			RM	
	Lost income costs for hospital visits [20]	€5.05/h of home help service	CM	NVH x TVH x UC
			RM	

^a^ Cost information was provided by the Cost Accounting Unit of Poniente Hospital

^b^ Data collected through questionnaires by participants in every visit to Poniente Hospital

^C^ CM = Conventional Monitoring; RM = Remote Monitoring

^d^ *NVH* number of visits to hospital; *PTH* time by physician per visit at hospital; *UC* unitary cost; *NRT* number of remote transfers to hospital; *PTR* time by physician per remote transfer; *ATH* time by administrative staff per visit at hospital; *ATR* time by administrative staff per remote transfer; *NVHA* number of visits to hospital by ambulance; *UCIT* unitary cost of informal transport; *TVH* unitary time spent by visit to hospital

From the PHS perspective, according to the labor agreement of Poniente Hospital, labor costs were divided taking into consideration the two staff categories, physicians and administrative staffs, involved in the study. Only direct electricity supply costs were included as the consultation-room costs; other indirect costs were ignored. As previous research [28], ambulance direct costs were also taken into consideration. Pacemaker recipient costs were divided equally for both the groups. The costs for the Medtronic CareLink® Network (RM group) were contributed by Medtronic© and thus free for the PHS and patients.

Transportation costs (taxi [32], bus [33] or private transport [34]), as well as the employment income loss for the time spent by patients and (in case) caregivers in every consultation visit [35], were considered from the patient’s perspective.

### Cost-utility analysis

The model compares intervention costs to clinical outcomes for each patient in terms of QALYs. QALY is a generic measure of disease burden, including both the quality and the quantity of life lived, used to assess the value of medical interventions. One QALY equates to 1 year in perfect health. In our study, the QALYs values were calculated by aggregating the utility scores of the *EuroQol–5D* (EQ–5D) Spanish validated version [36, 37] submitted by each patient at the end of the twelfth month (by 1 year) and fifth year (by 4 years), after applying the selected discount rate. However, due to temporary comparative reasons, the utility scores collected at the implantation date and after 6 months were ignored in this study. The health-related quality of life (HRQoL) in terms of utilities was measured by EQ–5D (from − 1 [the poorest imaginable health state] to 1 [perfect health]). The cost-utility ratio was evaluated by comparing differential QALYs and differential costs between CM and RM groups, after 5 years. The incremental cost-effectiveness ratio (ICER) was determined by calculating costs per QALYs gained for RM as compared to the CM group in the present study. According to the recommendations of the US Panel on Cost-Effectiveness in Health and Medicine [38, 39], future costs and QALYs were discounted by 3%.

### Primary model robustness analysis

The robustness of base-case results was evaluated by conducting a probabilistic sensitivity analysis (PSA) using Monte-Carlo simulation with 5000 iterations per group, as shown by existing research [40]. In PSA, uncertainty was evaluated by simultaneously varying stochastic variables in the Cost-per-patient model (Table 1) through random sampling from their assumed distributions (Table 2).

**Table 2:** Estimation of stochastic variables for Probabilistic Sensitivity Analysis

Variable^**a**^	Monitoring	Intervention groups [n (CM) = 34; n (RM) = 21]			Monte-Carlo simulation [n (CM) = 5000; n (RM) = 5000]			Assumed distribution
		Mean	Std.Dev.	Interval	Mean	Std.Dev.	Interval	
**NVH**	CM	7.49	1.70	(4–12)	9.40	7.24	(2–88)	Gamma
	RM	4.38	2.62	(2–10)	6.38	37.34	(2–30)	Gamma
**NRT**	CM	.	.	.	.	.	.	.
	RM	6.62	1.72	(4–9)	8.64	6.41	(2–48)	Gamma
**NVHA**	CM	1.91	0.70	(1–3)	1.90	1.80	(0–16)	Gamma
	RM	1.33	0.66	(0–3)	1.31	1.24	(0–10)	Gamma
**UCIT**	CM	8.72	9.31	(2.7–40.27)	8.69	3.18	(1.28–22.88)	Gamma
	RM	7.57	7.71	(2.7–34.56)	7.58	1.44	(3.55–13.36)	Gamma
**TVH**	CM	75.43	39.43	(0–180)	74.80	63.83	(0.33–495.32)	Gamma
	RM	88.57	44.98	(0–180)	88.56	76.33	(0.04–645.03)	Gamma
**EQ–5D M12**	CM	0.82	0.37	(−0.654–1)	0.81	0.73	(0–6.59)	Gamma
	RM	0.90	0.19	(0.362–1)	0.93	0.90	(0–7.65)	Gamma
**EQ–5D M60**	CM	0.77	0.36	(−0.572–1)	0.75	0.66	(0–5.62)	Gamma
	RM	0.68	0.39	(−0.33–1)	0.69	0.55	(0–4.28)	Gamma

^a^ *CM* Conventional Monitoring; *RM* Remote Monitoring; *NVH* number of visits to hospital; *NRT* number of remote transfers to hospital; *NVHA* number of visits to hospital by ambulance; *UCIT* unitary cost of informal transport; *TVH* unitary time spent by a visit to hospital; *EQ–5D M12* EuroQoL five-dimensional questionnaire after 12 months; *EQ–5D M60* EuroQoL five-dimensional questionnaire after 5 years

### Statistical analysis

Statistical analysis was conducted using SPSS (Statistical Package for Social Sciences) statistical software v.18.0.0 (SPSS Institute, Inc., Chicago, IL, USA). Differences between groups were compared using Mann-Whitney U-test for continuous independent and non-normally distributed variables. Results were presented, including the corresponding 95% confidence intervals (95% CI). Logistic regression was used to assess the influence of age, sex, and the method of monitoring attrition.

## Results

### Patient baseline characteristics

After the five-year follow-up period, 55 patients finished the PONIENTE trial (CM: 34; RM: 21) with a mean age of 81 ± 7 years. The female population of the patients comprised of 31%. Significant differences with regards to the assessed clinical characteristics were not observed between the study groups. As shown in Table 3, syncope was the most important disease manifestation (60%), and 47.27% of the patients experienced paroxystic Atrial Fibrillation episodes. The atrioventricular block was the most common pacing indication (71%), followed by sick sinus syndrome (20%).

**Table 3:** Patients’ characteristics

	All	RM group (***n*** = 21)	CM group (***n*** = 34)	***p***-value
Age (mean) ± SD	81.00 ± 6.47	81.14 ± 7.30	80.91 ± 6.01	*0.690*
Women (%)	17 (30.91)	8 (38.10)	9 (26.47)	*0.365*
Attrition (^a^)	28 (34.14)	9 (27.58)	18 (33.96)	*0.481*
***Pacing indication (%)(***^**b**^***)***				
Sinus node disease	11 (20.00)	3 (14.29)	8 (23.53)	*0.493*
Atrioventricular block	39 (70.91)	15 (71.43)	24 (70.59)	
Others	5 (9.09)	3 (14.29)	2 (5.88)	
***Disease manifestations (%)(***^**b**^***)***				
Syncope	33 (60.00)	13 (61.90)	20 (58.82)	*0.681*
Dizziness	16 (29.09)	7 (33.33)	9 (26.47)	
Dyspnoea	3 (5.45)	0 (0)	3 (8.82)	
Angina	3 (5.45)	1 (4.76)	2 (5.88)	

*CM* Conventional monitoring; *RM* Remote monitoring

(^a^) Concerned to the total initial patients (83)

(^b^) Indications and manifestations before the PM implant over the remaining sample at the end of the study

### Attrition

Owing to non-cardiovascular causes, 22 patients (RM: 8; CM: 14) died, whereas 6 patients (RM: 1; CM: 5) withdrew from the study. The loss of subjects at 5 years did not correlate with the sex (*p* = 0.910), the age of the participants (*p* = 0.880), or the study criteria variable, type of monitoring conducted (*p* = 0.110).

### Costs analysis

After the five-year follow-up period results in Table 4 revealed that total costs per patient were 23.02% lower for the RM group than the control group (CM), corresponding to a saving of €82.10 per patient (RM: €274.52 ± 128.45; CM: €356.62 ± 144.12; *p* = 0.033).

**Table 4:** Cost analysis

Perspectives	Variables	All			CM group			RM group			CM vs. RM Difference per patient	
		(***n*** = 55)			(***n*** = 34)			(n = 21)				
		Mean	SD	95% CI	Mean	SD	95% CI	Mean	SD	95% CI	Mean dif.	***p***-value
	*In-office visits per patient*	6.27	2.56	(5.58–6.97)	7.44	1.71	(6.84–8.04)	4.38	2.62	(3.19–5.57)	3.06	< 0.001
	*Transmissions from home per patient*	–	–	–	–	–	–	6.62	1.72	(5.84–7.4)	–	–
**Public Health System**	Physician (€)	157.50	46.80	(144.85–170.15)	164.72	37.83	(151.52–177.92)	145.82	57.62	(119.59–172.05)	18.90	0.191
	Administrative (€)	11.82	3.29	(10.93–12.71)	11.67	2.68	(10.74–12.61)	12.06	4.15	(10.18–13.95)	−0.39	0.703
	Staff costs (Physician + Administrative) (€)	169.32	49.97	(155.81–182.83)	176.39	40.51	(162.26–190.53)	157.88	61.75	(129.77–185.99)	18.51	0.231
	Transport in ambulance (€)	63.99	55.35	(49.03–78.96)	70.11	63.42	(47.98–92.24)	54.09	38.33	(36.64–71.54)	16.01	0.248
	Consultation room (€)	5.75	2.54	(5.07–6.44)	7.14	1.64	(6.57–7.71)	3.50	2.09	(2.55–4.46)	3.64	< 0.001
	Total PHS costs (€)	239.07	94.79	(213.44–264.69)	253.64	95.86	(220.19–287.08)	215.48	90.28	(174.38–256.57)	38.16	0.144
**Patients and relatives**	*Distance home/hospital (one way kms)*	16.25	11.50	(13.15–19.36)	15.71	11.71	(11.62–19.79)	17.14	11.37	(11.97–22.32)	−1.44	0.657
	Informal transport (€)	32.75	39.81	(21.99–43.52)	42.42	46.99	(26.02–58.81)	17.11	14.81	(10.37–23.85)	25.31	0.006
	Lost income (€)	53.45	35.43	(43.88–63.03)	60.57	34.45	(48.55–72.59)	41.94	34.73	(26.13–57.75)	18.63	0.059
	Total patients’ costs (€)	86.21	57.16	(70.76–101.66)	102.98	58.77	(82.48–123.49)	59.05	43.24	(39.36–78.73)	43.93	0.002
**Total Costs**		325.28	142.91	(286.64–363.91)	356.62	144.12	(306.34–406.91)	274.52	128.45	(216.06–332.99)	82.10	0.033

*CM* Conventional monitoring; *RM* Remote monitoring

Values are expressed as means and standard deviation (SD) (95% CI)

Costs are concerned to the total 5-years follow up period. 3% discount rate applied to annual costs

#### Public health system perspective

Significant differences between the mean in-hospital visits per patient of CM and RM groups were estimated after the five-year follow-up period. The number of all types of visits (scheduled or not) to the hospital was higher in the CM group (7.44 ± 1.71) as compared to the RM group (4.38 ± 2.62) (*p* < 0.001).

Regarding labor costs, nonsignificant differences were recorded for RM group against CM group (RM: €157.88 ± 61.75; CM: €176.39 ± 40.51; *p* = 0.231). Staff cost differences were nonsignificant regarding physician labor costs (RM: €145.82 ± 57.62; CM: €164.72 ± 37.83; *p* = 0.191) and administrative staff costs (RM: €12.06 ± 4.15; CM: €11.67 ± 2.68; *p* = 0.703). The former revealed that physicians, unlike administrative staff, spent more time per patient in the CM group, representing a mean €18.90 cost saving per patient.

Lower usage of transport via ambulance was also revealed by the RM group (RM: €54.09 ± 38.33; CM: €70.11 ± 63.42; *p* = 0.248). Consultation room costs were also lower in the RM group (*p* < 0.001).

In total, the RM group exhibited a cost saving of 15.04% per patient from the PHS perspective without statistical significance (RM: €215.48 ± 90.28; CM: €253.64 ± 95.86; *p* = 0.144).

#### Patients’ perspective

Although no statistical significance in terms of distance from home to hospital (*p* = 0.657) was found between RM and CM groups (one-way trip was lower in CM group 15.71 ± 11.71 km than in the RM group 17.14 ± 11.37 km), results showed a significant reduction of travel cost for patients in RM group (€17.11 ± 14.81) in comparison to CM group (€42.42 ± 46.49) (*p* = 0.006). Results also portrayed cost saving in the RM group as compared to CM group in terms of income loss of patients and caregivers in every in-office visit (RM: €41.94 ± 34.73; CM: 60.57 ± 34.45; *p* = 0.059). Overall, from the patients’ perspective, RM implied a cost saving of 42.66% (RM: €59.05 ± 43.24; CM: €102.98 ± 58.77; *p* = 0.002).

### Cost-utility analysis

As shown in Table 5, statistically nonsignificant differences were revealed between groups alongside the five-year follow-up period.

**Table 5:** Utility values, quality-adjusted life years (QALYs), and cost per patient along the five-year study period

Variables	All			Conventional Monitoring group			Remote Monitoring group			Difference per patient	
	n	Mean	SD	n	Mean	SD	n	Mean	SD	Mean dif.	***p***-value
EQ5 DM00	55	0.71	0.36	34	0.72	0.36	21	0.70	0.38	0.02	0.795
EQ5 DM12	55	0.85	0.32	34	0.82	0.37	21	0.90	0.19	−0.08	0.301
EQ5 DM60	55	0.73	0.37	34	0.77	0.36	21	0.68	0.39	0.09	0.363
QALYM60	55	3.48	1.47	34	3.58	1.45	21	3.31	1.52	0.27	0.515
Cost/QALYM60 (€)	55	195.64	515.73	34	177.08	357.90	21	225.69	710.80	−48.61	0.773
Cost/patient (€)	55	325.28	142.91	34	356.62	144.12	21	274.53	128.45	82.10	0.033
ICER	–	–	–	–	–	–	–	–	–	301.16	–

*EQ–5D M12* EuroQoL five-dimensional questionnaire after 12 months; *EQ–5D M60* EuroQoL five-dimensional questionnaire after 5 years; *QALYM60* quality adjusted life years after 5 years; *ICER* incremental cost-effectiveness ratio (ICER), calculated as the difference in the expected cost per patient (€) divided by the difference in the QALY produced by the two interventions (CM and RM)

3% discount rate applied to annual costs and QALYs

**Fig. 1:**
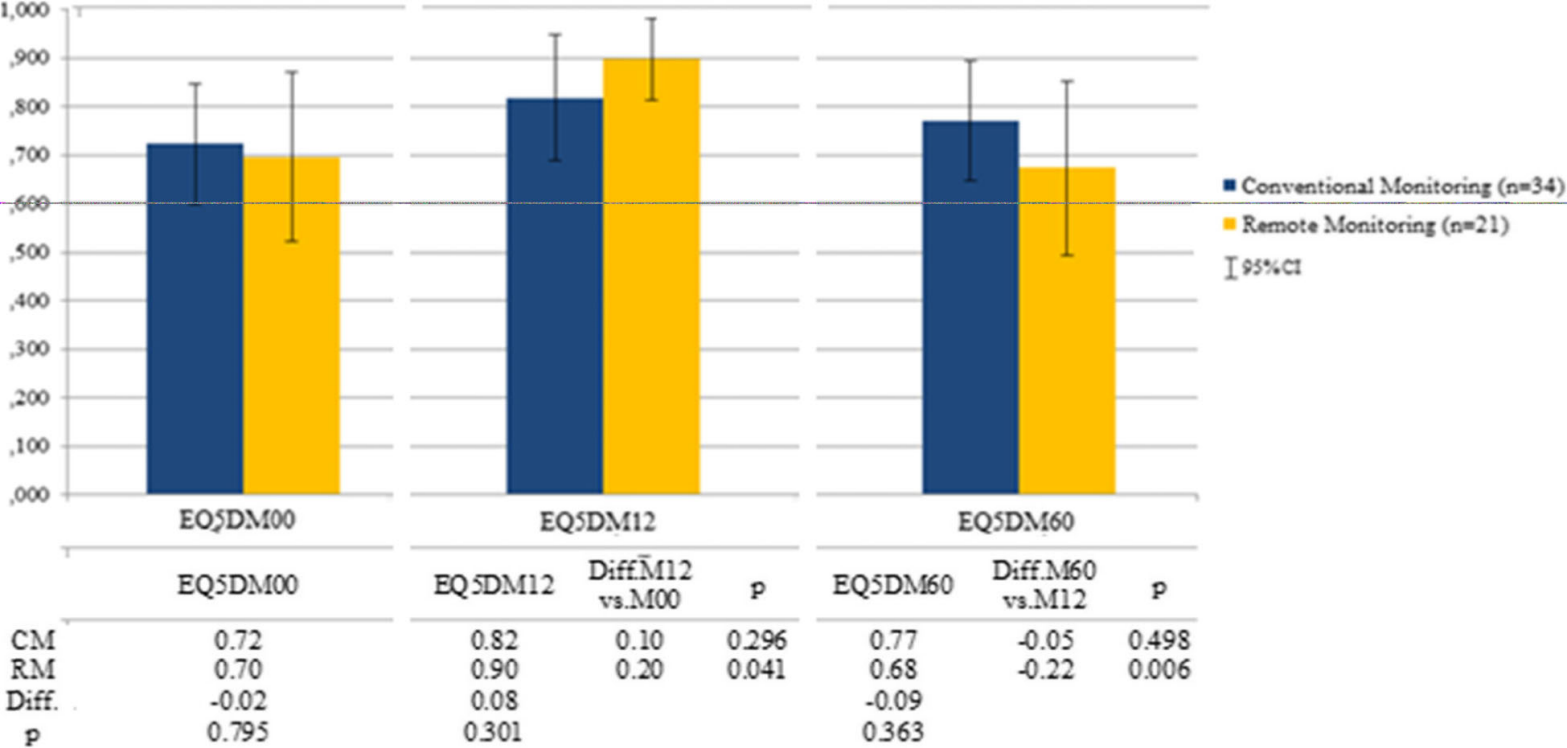
Evolution of Utilities along the five-year follow-up period

At the time of enrolment in the study, the overall mean EQ–5D questionnaire of health-related quality of life scores in utilities showed better for patients in the CM group (EQ5DM00 = 0.72) in comparison to RM group (EQ5DM00 = 0.70). After the first 12-months follow-up period, figures became better in the RM group (EQ5DM12 = 0.90) than in the CM group (EQ5DM12 = 0.82). However, at the end of the study, after 5 years months of follow-up, the reduction was evident in the overall mean scores for the RM group (EQ5DM60 = 0.68) than the CM group (EQ5DM60 = 0.77). The functional capacity scores were discreetly maintained or slightly declined at 5 years after implantation. Evolution of EQ–5D utilities along the study was depicted in Fig. 1.

Costs/QALYs obtained at 5 years follow up by the RM group (€225.69) were higher than the CM group (€177.08), although this data was nonsignificance statistically (*p* = 0.773). Patients belonging to the CM group manifested a slightly better QALY at the end of the study (3.58) than the RM group (3.31). Regarding that, the RM group reported an €82.10 cost-saving per patient (*p* = 0.033), the ICER of CM in comparison to RM became positive (€301.16) as a measure of cost-effectiveness of RM in contrast to CM (Table 5).

### Primary model robustness analysis

The results of the robustness test in Table 6 were in accordance with the data obtained in the previous sections. Statistical analysis confirmed the overall costs per patient in RM group was significantly lower than the CM group, corresponding to a €53.68 cost saving per patient (RM: €384.92 ± 187.35; CM: €438.60 ± 324.29; *p* < 0.001). Significant statistical difference was, however, lacking in the labor costs between RM and CM groups (RM: €221.70 ± 99.03; CM: €222.70 ± 171.67; *p* = 0.720) as a consequence of the effect of physician labor costs (RM: €204.92 ± 91.68; CM: €207.96 ± 160.31; *p* = 0.244) and a contradictory effect of administrative staff costs (RM: €16.78 ± 7.47; CM: €14.74 ± 11.36; *p* < 0.001). Transportation by ambulance, as well as consultation room costs, was lower in the RM group. Therefore, the RM group saved a cost of 4.50% per patient from the PHS perspective (RM: €288.36 ± 134.59; CM: €301.95 ± 201.34; p < 0.001). Moreover, from patients’ perspective also a significant cost saving of RM group was confirmed by the robustness analysis (RM: €96.56 ± 86.20; CM: €136.64 ± 153.12; p < 0.001), as a consequence of a significant difference in travel cost in RM group as well as costs-saving in the RM group in comparison to CM group with respect to income loss of older adults patients and caregivers in every in-office visit.

**Table 6:** Cost analysis–robustness test

Perspectives	Variables	All			CM group			RM group			CM vs. RM Difference per patient	
		(***n*** = 10,000)			(***n*** = 5000)			(***n*** = 5000)				
		Mean	SD	95% CI	Mean	SD	95% CI	Mean	SD	95% CI	Mean dif.	***p***-value
**Public Health System**	Physician (€)	206.44	130.58	(203.88–209)	207.96	160.31	(203.52–212.41)	204.92	91.68	(202.37–207.46)	3.05	0.244
	Administrative (€)	15.76	9.67	(15.57–15.95)	14.74	11.36	(14.42–15.05)	16.78	7.47	(16.57–16.99)	−2.04	< 0.001
	Staff costs (Physician + Administrative) (€)	222.20	140.13	(219.45–224.95)	222.70	171.67	(217.94–227.46)	221.70	99.03	(218.95–224.44)	1.00	0.720
	Transport in ambulance (€)	65.90	88.92	(64.16–67.64)	70.24	88.88	(67.77–72.7)	61.56	88.75	(59.1–64.02)	1.78	< 0.001
	Consultation room (€)	7.06	5.67	(6.95–7.17)	9.02	6.95	(8.82–9.21)	5.10	2.87	(5.02–5.18)	3.91	< 0.001
	Total PHS costs (€)	295.16	171.38	(291.8–298.52)	301.95	201.34	(296.37–307.53)	288.36	134.59	(284.63–292.09)	13.59	< 0.001
**Patients and relatives**	Informal transport (€)	47.86	54.14	(46.8–48.92)	60.64	69.11	(58.72–62.55)	35.08	27.56	(34.31–35.84)	25.56	< 0.001
	Lost income (€)	68.74	88.61	(67.01–70.48)	76.01	103.28	(73.14–78.87)	61.48	70.25	(59.53–63.43)	14.53	< 0.001
	Total patients’ costs (€)	116.60	125.85	(114.13–119.07)	136.64	153.12	(132.4–140.89)	96.56	86.20	(94.17–98.95)	40.09	< 0.001
**Total Costs**		411.76	266.17	(406.54–416.97)	438.60	324.29	(429.6–447.59)	384.92	187.35	(379.72–390.11)	53.68	< 0.001

*CM* Conventional monitoring; *RM* Remote monitoring

Values are expressed as means and standard deviation (SD) (95% CI)

A positive ICER of CM in comparison to RM (€501.48) affirmed by the cost-utility robustness analysis (Table 7) opined that RM remained a cost-utility alternative in comparison to CM. As in thse primary model, slightly better QALY figures for patients in the CM group (CM: 3.48 ± 2.48; RM: 3.38 ± 2.19; *p* = 0.022) were compensated by a cost per patient reduction in the RM group. Moreover, unlike the primary model, Costs/QALYs obtained by the RM group (€192.43) were significantly (*p* < 0.001) lower than the CM group (€238.73), as computed by statistical analysis.

**Table 7:** Cost-utility analysis–robustness test

Variables	All			Conventional Monitoring (CM) group			Remote Monitoring (RM) group			Difference per patient	
	(***n*** = 10,000)			(***n*** = 5000)			(***n*** = 5000)				
	n	Mean	SD	n	Mean	SD	n	Mean	SD	Mean dif.	***p***-value
EQ5 DM12	10,000	0.87	0.82	5000	0.81	0.73	5000	0.93	0.90	−0.12	< 0.001
EQ5 DM60	10,000	0.72	0.61	5000	0.74	0.66	5000	0.69	0.55	0.06	< 0.001
QALYM60	10,000	3.43	2.34	5000	3.48	2.48	5000	3.38	2.19	0.10	0.022
Cost/QalyM60 (€)	10,000	215.58	543.95	5000	238.73	721.12	5000	192.43	265.94	46.30	< 0.001
Cost/patient (€)	10,000			5000	438.60	324.29	5000	384.92	187.35	53.68	< 0.001
ICER	–	–	–	–	–	–	–	–	–	501.48	–

## Discussion

### Main findings

After the five-year follow-up period, cost differences found between RM and CM groups became irrelevant but significant, reporting an €82.10 cost-saving per older adults patient in the RM group (*p* = 0.033) in the total 5-years follow up period. At the end of the study, the CM group showed a better QALY (3.57 vs. 3.30). This result was confirmed by the robustness test. A lower cost per QALY ratio in the RM group (€192.43 vs. €238.73) was also observed. Subsequently, a positive incremental cost-effectiveness ratio (ICER) of CM in comparison to RM was found (€301.16).

The mean number of scheduled and unscheduled in-hospital visits per patient was reduced by 41.52% for the RM group, in line with existing research [14, 27, 41, 42]. These outcomes reinforced the results obtained in the 12-months PONIENTE study [30] that showed a reduction of in-hospital visits by 26.89%. Moreover, more recent research [11] confirmed this finding after evaluating a reduction of 79.20% in the number of in-office visits for 445 patients with PMs and Implantable Cardioverter Defibrillator (unlike our study that only focuses on patients with PMs) followed-up during more than 24 months through RM plus remote interrogations every 6 months. This reduction incurred a significant cost saving of 44.88% from the patients’ perspective, diminishing travel cost for the older adults as well as the level of income loss of patients and caregivers in the RM group.

However, after a five-year follow-up period, this reduction of in-hospital visits failed to display a significant impact on the costs from the PHS perspective, with a cost saving of 16.13% per patient (€215.48 vs. €256.92; *p* = 0.112). Although a significant reduction in costs from the PHS perspective of 57.64% in the RM group (*p* < 0.001) was reported in our 12-months PONIENTE study [29]. This finding was in accordance with the previous studies [5, 10, 25, 27, 43–45]. After 5 years, the management costs of patients under RM seemed to be unbalanced by the incremental burdens of following up automatic wireless remote notifications by physicians and administrative staff. More recent pacemakers technology is presented as opportunities to allow constant patient “big data” collection, to optimize the outcomes of patients. Henceforth, for future research on remote monitoring, this should be taken into consideration. Thus, as recently suggested, including higher survival ratios in comparison to periodic in-hospital evaluations may be achieved through more frequent (even daily) RM transmissions [46]. However, higher efforts must still be made by the clinicians to enable a more efficient practice. With the aim to prioritize clinical decisions and interventions as well as to reduce practitioners’ burdens automatized RM data triage through artificial intelligence methods [46] may be implemented.

Although the 12-months PONIENTE study showed an increment in QALYs (+ 0.09) among older patients with PMs under RM as compared to those under CM, our results recorded a higher QALYs decrease (− 0.20) for the RM group after the five-year follow-up period. On the contrary, a negative effect on the patients’ quality of life was not published by other studies, although for shorter follow-up periods (maximum 24 months) [25, 47, 48] even by using other questionnaires (i.e., SF–36 questionnaire) [10]. These results may indicate a different evolution of utilities in patients under RM for a longer period. Published reports in the literature revealed long-term undesirable effects on the quality of life of patients with pacemakers implantation under conventional monitoring [49]. This previous study observed a moderate increase in the SF–36 general scores for patients with bradycardia pacemaker implantation for the first 24 months post-implantation, which was followed by a gradual decline over time. Physical reasons were the causes of this gradual decline, unlike mental scores, as a consequence of non-cardiovascular comorbid diseases usual among geriatric patients. Thus, persistent increase in mental scores drawn from emotional problems and social dysfunctions reflected improved well-being after PM implantation [49]. According to that, some of the major RM benefits from patients’ perspectives drawn from the reduction of their in-hospital visits may not be appreciated in the long term in comparison to CM. Moreover, this indicated the need for better patient education regarding the purpose and benefits of RM, its usability, and safety [16], contrary to the findings in short-terms remote monitoring studies [25]. This leads us to the wide debate around social acceptance of telehealth. For shorter follow-up periods, some research does state that a well-structured tele-medical centre available 24 h a day to act promptly according to the individual patient’s risk profile in addition to educational activities [50], as well as continuous ‘remote’ contact with patients sending clinical reports [11], contribute to balance patients’ acceptance. Other studies have found that patients may prefer an in-hospital consultation to establish a diagnosis or to plan their treatment [19]. In the long term this situation may be assimilated to the follow-up of patients with PM. However, to our knowledge, this is the first study where the EQ–5D questionnaire has been used to assess the HRQoL in older patients with PMs under RM for a long-term follow-up period of 5 years. Thus, QALYs obtained could not be compared with those in previous papers on the RM of users with PMs.

Regarding both, PHS and older patients’ perspective, cost-utility analysis confirmed that after a 5-years follow-up period, slightly better QALY figures for patients in the CM group may be compensated by a cost per patient reduction in the RM group, assessing in €301.16 the ICER of CM in comparison to RM.

### Limitations

Although no statistically significant differences were found between both groups in baseline variables assessed (Table 3), the first methodological limitation lies in the fact that it is a non-randomized study where the type of monitoring was recommended by older patients together with the physicians, according to the patients’ characteristics. However, no effect of other essential variables on the outcomes was found by the propensity score matching, conducted to explore the variables influencing the election of the type of monitoring [6, 30]. In any case, greater external validity seems to be achieved as the assignment form used fits the daily practice, and probably the outcomes will be closer to those expected in the real world [51, 52]. Furthermore, the basal characteristics studied were very similar to those reported in the Spanish Pacemaker Registry [53], which supported its generalization. Secondly, it is an open trial where the type of monitoring for every patient was known by both the patients and physicians. This might indulge in biasness in the patients’ and physicians’ behavior. Third, other decives more than pacemakers, like implantable cardioverter defibrillators (ICD) or cardiac resynchronization therapy devices (CRT), have not been inserted in this study. Then, our results can not be extended to complete devices patients. Fourth, differences of sample size among groups, as well as the limited population size drawn from a single center with a limited number of implants per year, might contribute to the reduction in the statistical power of the study. The similarity of the results with existing research [3, 5, 8, 14, 15] does not suggest the influence of differences in the size of both groups on our findings. The level of attrition along the five-year follow-up period may also have affected the results. However, the analysis of attrition demonstrated the similarity between participants who left and those who remained in the program for both groups of monitoring. In any case, as far as the loss of subjects is concerned, the methodology followed in this study prevented generalization of the results among non-survivors. In the same way, generalization among other European Health Public Systems should be cautious as the sample was concentrated in a single Spanish hospital. Finally, the final cost difference between both groups may be affected by the fact that the CareLink Monitor associated cost was afforded by the supplier (Medtronic©), so it was not considered within either the PHS or the patients’ costs.

## Conclusions

The PONIENTE trial affirmed, after 5 years of RM of older patients with pacemakers, that it remains a cost-utility alternative to conventional monitoring in hospitals. However, although significant cost savings were still appreciated from older patients’ perspective, PHS management costs differences between both groups seemed to be leveled off. Optimization of the incremental staff burdens of following up automatic remote notifications is presented as a challenge for future studies in RM of older patients with pacemakers.

## Data Availability

The datasets used and/or analyzed during the present study are available from the corresponding author on reasonable request.

## Abbreviations

RM: Remote Monitoring
CM: Conventional Monitoring
QALYs: Quality-Adjusted Life Years
PHS: Public Health System
PM: Pacemakers
EQ–5D: *EuroQol–5D*
HRQoL: Health-Related Quality of Life
ICER: Incremental Cost-Effectiveness Ratio
